# Supplementary material for: Geographical characteristics and formation mechanisms of smallpox epidemics in Hubei Province, China, 1488–1949
Source: PLoS One. 2025 May 13;20(5):e0317108. doi: 10.1371/journal.pone.0317108 (PMC12074586; doi:10.1371/journal.pone.0317108)
Supplement: Attachment — Submitted filename: 2024.10.24Response to Reviewers.docx [file pone.0317108.s003.docx]

**Response to the Reviewers**

We are very grateful to the two reviewers for their valuable comments. We have made detailed revisions based on their comments, which have been of great help in improving the quality of this article. The following is a point-by-point response to the reviewers’ comments.

**Reviewer #1: The provided research paper was an excellent attempt from the authors point of view small pox epidemic in Hubei provience,China but required some modification in the relevant literature with proper standarized reference style. In addition, the methods section must be modify with organometric and systematic proper mechanism and formation of epidemic pattern of small pox variation with chronological form.**

Thanks to the reviewers' comments, we have added some new literature and revised the reference format according to the standarized reference style. Regarding the research methods, the current research methods are introduced from three aspects: temporal changes (trend cycle analysis methodology), spatial distribution (spatial autocorrelation model, epidemic center of gravity model), and formation mechanism (geographically weighted regression model), which are consistent with the research content and sequence of this article.

Reviewer #2: This article is written well with statistical data analysis. How ever, a few correction should be addressed by authors.

**1. Please update data set up to date.**

We have uploaded the original maps, tables, and analysis results as Supporting Information files.

**2. Clear research place of interest.**

We re-summarized the research place of interest.

What are the spatiotemporal characteristics of the epidemic of smallpox in Hubei history? What are the influencing factors and formation mechanisms of its spatiotemporal characteristics? These are the questions we are interested in.

**3. Why one M-K test is used without others. It would be better if authors use more model and compare them with validation. In this case Machine learning will be the best.**

Thank you for the tips on research methods. We have tried wavelet analysis, machine learning and other methods to analyze the time trend of smallpox, but overall, the MK test is more suitable. In addition to detecting mutation points, it does not require high data quality, is still effective when the amount of data is small, and the overall analysis results are not affected by a few outliers. In subsequent research, we will try other machine learning models to find the most appropriate time series analysis method.

**4. River networks, road networks, war, and other factors promoted smallpox epidemics. These are already established. Authors need to add more result-based evidence and data analysis to find out new outputs.**

Based on the geographically weighted regression analysis model, this article analyzes the main influencing factors of smallpox and finds that factors such as river networks, road networks, and wars have a promoting effect on the epidemic of smallpox. In addition, based on the analysis of the regression coefficients of each factor, we can know the regional differences in the degree of influence of these influencing factors. For example, the regression coefficients of the river network density and the number of war years are both positive and both show a "low in the west and low in the east" The spatial distribution characteristics of "high" indicate that river networks and war factors have a promoting effect on the epidemic of smallpox, and are more significant in eastern Hubei. These are some new findings of this article.

**5.Abstract:**

**Conclusion needs to be edited as per main findings and its implications.**

We have edited the conclusion section based on the main findings.

(1) From the perspective of temporal changes, Hubei smallpox may be traced back to the Ming Dynasty. There are still few smallpox records from the Qing Dynasty. There were 9 smallpox years in the 268 years of the Qing Dynasty (1644-1911), for a frequency of 3.4%. In the Republic of China (1912-1949), there were 36 smallpox epidemics that occurred over 38 years, for a frequency of 94.74%. During the Republic of China period, smallpox showed a downward trend between 1912 and 1931, and an upward trend between 1931 and 1949. 1939 was the time when the smallpox epidemics began to suddenly increase.The smallpox epidemics in the Republic of China had two time scale fluctuation cycles: 8 years and 18 years. The main seasons for smallpox epidemics are spring and summer, followed by winter, and the least common is autumn.

(2) From the perspective of spatial distribution, smallpox epidemics in Hubei Province had a wide spatial scope and simultaneously showed the characteristics of spreading and proliferation; three towns of Wuhan and the Suixian and Yichang lines were the epidemic centers in the eastern, northern and western regions of Hubei, respectively. Smallpox epidemics in Hubei Province exhibited a significant spatial clustering pattern, with high and high clustering areas mainly distributed in Wuchang, Hankou and Hanyang; high and low clustering areas in Suixian in northern Hubei; and low and high clustering areas in Jiayu and Huangpi; these regions were distributed around the three towns of Wuhan and did not appear in the low and low clustering areas. The center of gravity of smallpox in Hubei Province exhibited a small swing from east to west in the central region and gradually shifted to the west, reflecting the gradual spread of the epidemic to the west and worsening of the disease.

(3) From the perspective of influencing factors and formation mechanisms, factors such as river networks, road networks, and wars all promoted smallpox epidemics. Among these factors, river network and war factors were significant in eastern Hubei, and road network factors were significant in southern Hubei. Therefore, drought in western Hubei had a certain inhibitory effect on smallpox epidemics. Temperature fluctuations, droughts and floods, and outbreaks of war played leading roles in the temporal variation characteristics of smallpox epidemics in Hubei Province. Lower temperatures and increased fluctuations in spring led to increases in epidemics. Frequent droughts and floods in summer often indirectly trigger smallpox epidemics. The outbreak of large-scale wars would push smallpox epidemics to peaks. Topography, population distribution and population mobility played dominant roles in the spatial distribution pattern of smallpox epidemics in Hubei Province, with topography revealing the basic pattern of smallpox distribution in Hubei Province, population distribution determining the overall pattern of smallpox distribution in Hubei Province, and population mobility reshaping the pattern of smallpox distribution in Hubei Province.

**6.Introduction:**

**Please clear research gaps properly.**

We re-examined the existing research and summarized the research gaps.

In summary, the existing studies mainly have the following deficiencies: First, most of them are qualitative studies on the spatiotemporal distribution, influencing factors, and response impacts of smallpox epidemics from a historical perspective, lacking comprehensive quantitative studies from the perspectives of geography, history, and epidemiology. In particular, the analysis of the influencing factors of smallpox is not systematic enough and lacks exploration of the formation mechanism; second, there is still a lack of specialized research on historical smallpox in Hubei, and existing research mainly focuses on cholera and malaria.

**Authors need to write about virus also.**

We have added a brief introduction to the smallpox virus.

Smallpox virus belongs to the family Poxviridae, genus Orthopoxvirus, which is susceptible to humans, mainly through airborne transmission, with high infectiousness and mortality.

**7.Methods:**

**Please add latitude and longitude.**

We have added latitude and longitude in Fig.1 Administrative division map of Hubei Province in 1946.

**Data files might be added in the supplementary files.**

We have added data files in the supplementary files, incluing original maps, tables, and analysis results.

**8.Results:**

**Please add the probable reasons for epidemiological profile changes in Hubei Province, specifically, the scenario of 1940.**

We have added the probable reasons for epidemiological profile changes in Hubei Province.

The smallpox epidemic in Hubei Province showed an overall trend of "light at the beginning and heavy at the end" in terms of time changes, mainly concentrated in the Republic of China period, especially in the 1940s. Overall, it was mainly caused by the frequent wars and floods and droughts during this period.

**4-5 figures are suggested, please move to supply file or merge with one another.**

We have moved the following figues to supply file:

Fig.5 Wavelet variance plot of the smallpox county series in Hubei Province

Fig.6 Changes in the real part of the wavelet transform coefficients of the smallpox county series at different time scales in Hubei Province

Fig.10 e.number of years of flooding, f.population density
