# Supplementary material for: Geographical characteristics and formation mechanisms of smallpox epidemics in Hubei Province, China, 1488–1949
Source: PLoS One. 2025 May 13;20(5):e0317108. doi: 10.1371/journal.pone.0317108 (PMC12074586; doi:10.1371/journal.pone.0317108)
Supplement: Attachment — Submitted filename: 2024.12.18Response to Reviewers.docx [file pone.0317108.s004.docx]

**Response to the Reviewers**

**We are very grateful for the valuable comments from the reviewers. We have made corresponding modifications and improvements based on them.**

**Reviewer #1**

The manuscript provides a comprehensive analysis of the spatial and temporal distribution of smallpox epidemics in Hubei Province, China, from 1488 to 1949. The study employs rigorous methodologies, including the M-K test, wavelet analysis, spatial autocorrelation, and geographically weighted regression models, to identify key environmental and social factors that influenced epidemic patterns. The historical insights and advanced statistical tools applied make this a valuable contribution to understanding epidemic patterns in historical contexts.

Suggested Revisions:

**1. Clarity in Methodology: While the methodology is sound, further clarification on certain models, such as the specifics of the wavelet analysis and M-K test implementation, would enhance reproducibility for readers unfamiliar with these techniques.**

In the research methods section, we supplemented the formulas, parameters, implementation tools, etc. of methods such as M-K test, wavelet analysis, spatial autocorrelation, epidemic center of gravity model, and geographically weighted regression analysis.

**2.Interpretation of Results: The section on the spatial shift of the epidemic center (east to west) could benefit from a deeper analysis of underlying socio-political or environmental factors to better contextualize this finding.**

We have supplemented the political and military factors that led to the migration of the epidemic center. In the 1930s and 1940s, the continuous outbreak of wars prompted a large number of military and political institutions and schools to migrate to Enshi in western Hubei Province, with a population of about 100,000, making Enshi the political, military and cultural center of Hubei Province. This was also an important reason for the migration of the center of gravity of smallpox from east to west.

**3.Discussion on Prevention and Control Implications: The conclusion briefly mentions preventive measures against smallpox and similar viruses. Expanding on this, particularly with lessons learned from historical epidemics, could provide valuable insights for modern epidemic management.**

In the part about prevention and control measures, based on the research on smallpox in Hubei, we summarized the preventive measures from three aspects: factors affecting temporal changes, factors affecting spatial distribution patterns, and drugs for smallpox prevention and treatment, and added content on preventing bioterrorism.

We must remain vigilant against bioterrorism, strengthen the management of smallpox virus, prevent smallpox virus from becoming a biological weapon, and conduct epidemic prevention exercises to respond to smallpox outbreaks.

**4.Conclusion: This manuscript is a valuable addition to historical epidemiological studies and enhances understanding of smallpox spread dynamics in historical China. With minor revisions to methodology and discussion, this paper would be a strong candidate for publication.**

Based on the previous suggestions, we have revised and improved the research methods and discussion sections.

**Reviewer #2**

**1.Abstract**

**Please mention data collection time and source.**

**Please input your significant results in abstract.**

We supplemented the data sources and collection time in the data collection section, and supplemented the content about smallpox prevention measures in the summary section.

**2.Introduction**

**Please add references: Smallpox originally originated in ancient Egypt or India 3,000 years ago.**

**(https://doi.org/10.1016/j.imj.2023.11.001)**

**Please add research gap regarding the issues.**

**Add more epidemiology from. https://doi.org/10.22207/JPAM.16.SPL1.16; https://doi.org/10.3390/ijerph192315638**

We added the relevant references and research conclusions.

[3]Tegnell A , Wahren B , Elgh F .Smallpox—eradicated, but a growing terror threat.Clinical Microbiology and Infection, 2002, 8( 8):504-509. doi:10.1046/j.1469-0691.2002.00525.x.

[39]Islam MA, Sangkham S, Tiwari A, et al. Association between Global Monkeypox Cases and Meteorological Factors. International Journal of Environmental Research and Public Health. 2022; 19(23):15638. doi: 10.3390/ijerph192315638.

[45]Islam MA, Mumin J, Haque MM, etl. Monkeypox virus (MPXV): A Brief account of global spread, epidemiology, virology, clinical features, pathogenesis, and therapeutic interventions. Infect Med (Beijing). 2023;2(4):262-272. doi:10.1016/j.imj.2023.11.001. PMID: 38205182; PMCID: PMC10774656.

**3.Methodology:**

**Justification is required for using Mann-Kendall (M-K) tests, wavelet analysis, and geographically weighted regression (GWR) and parameter selection for these methods, such as the bandwidth used in GWR and justification for specific thresholds in the M-K test.**

**Manuscript used multiple data sources however there is lack of clarity on data integration techniques.**

**Please write on data cleaning and processing.**

In the research methods section, we supplemented the formulas, parameters, implementation tools, etc. of methods such as M-K test, wavelet analysis, spatial autocorrelation, epidemic center of gravity model, and geographically weighted regression analysis.

The smallpox epidemics data and influencing factors data were unified and integrated through data standardization, with years as the basic time unit and counties as the basic spatial unit.

**4.Result**

**Please add why some regions or time periods showed higher or lower smallpox incidence.**

**Moran’s I and local Moran’s I findings showed statistically significant. Pleas add more practical implications.**

In the part of formation mechanism,we analyzed the main factors affecting the temporal variation and spatial distribution of smallpox.Temperature fluctuations, droughts and floods, and war outbreaks played dominant roles in the temporal characteristics, for example, smallpox in Hubei Province was prevalent mainly in spring and summer, primarily because the temperature in spring is low, which is suitable for the survival of smallpox virus, and fluctuates greatly, which reduces human immunity and increases the number of susceptible people. The topography, population distribution and population movement played dominant roles in the spatial distribution pattern. For example, in the 1930s and 1940s, the continuous outbreak of wars prompted a large number of military and political institutions and schools to migrate to Enshi in western Hubei Province, with a population of about 100,000, making Enshi the political, military and cultural center of Hubei Province. This was an important reason for the migration of the center of gravity of smallpox from east to west.

We added the practical implications of the Moran’s I and local Moran’s I findings. The distribution of smallpox epidemic in Hubei Province showed a clustered state. High-high clusters mainly distributed in Wuchang, Hankou, and Hanyang, which indicates these places were the hardest hit areas and high-risk areas of smallpox in Hubei Province. High-low clusters distributed only in Suixian, which located in the northern region of Hubei. Low-high clusters distributed in Jiayu and Huangpi, mainly in the areas around the three towns of Wuhan. There was no low-low clusters.

**Reviewer #3**

Accept
